# Visceral fat dominant distribution in male type 2 diabetic patients is closely related to hepatic insulin resistance, irrespective of body type

**DOI:** 10.1186/1475-2840-8-44

**Published:** 2009-08-05

**Authors:** Yoshinori Miyazaki, Ralph A DeFronzo

**Affiliations:** 1 Department of Medicine, Diabetes Division, University of Texas Health Science Center, San Antonio, TX, USA; 2 2nd Department of Internal Medicine, Sapporo Medical University School of Medicine, Sapporo, Japan

## Abstract

**Background:**

All previous studies that investigated the association between abdominal fat distribution and insulin resistance evaluated subcutaneous and visceral fat area and/or volume, but these values were not related to the body type of each subject. In the present study we have examined the association between abdominal fat distribution and peripheral (muscle)/hepatic sensitivity to insulin using the visceral to abdominal subcutaneous fat area ratio (VF/SF ratio) in male patients with type 2 diabetes mellitus. This ratio defines the predominancy of visceral or subcutaneous abdominal adiposity, independent of the body type of each individual.

**Methods:**

Thirty-six type 2 diabetic male patients underwent a euglycemic insulin clamp (insulin infusion rate = 40 mU/m^2^·min) with 3-^3^H-glucose to measure insulin-mediated total body (primarily reflects muscle) glucose disposal (TGD) and suppression of endogenous (primarily reflects liver) glucose production (EGP) in response to a physiologic increase in plasma insulin concentration. Abdominal subcutaneous (SF) and intraabdominal visceral fat (VF) areas were quantitated with magnetic resonance imaging (MRI) at the level of L_4–5_.

**Results:**

TGD and TGD divided by steady state plasma insulin concentration during the insulin clamp (TGD/SSPI) correlated inversely with body mass index (BMI), total fat mass (FM) measured by ^3^H_2_O, SF and VF areas, while VF/SF ratio displayed no significant relationship with TGD or TGD/SSPI. In contrast, EGP and the product of EGP and SSPI during the insulin clamp (an index hepatic insulin resistance) correlated positively with VF/SF ratio, but not with BMI, FM, VF or SF.

**Conclusion:**

We conclude that, independent of the individual's body type, visceral fat dominant accumulation as opposed to subcutaneous fat accumulation is associated with hepatic insulin resistance, whereas peripheral (muscle) insulin resistance is more closely related to general obesity (i.e. higher BMI and total FM, and increased abdominal SF and VF) in male patients with type 2 diabetes.

## Background

Reduced insulin-mediated glucose disposal in muscle and impaired suppression of hepatic glucose production by insulin are common metabolic features of both obesity and type 2 diabetes mellitus (T2DM) [1]. A close association between obesity and T2DM is well established [2,3]. Many studies have documented that intraabdominal visceral fat is closely associated with insulin resistance in obese non-diabetic and T2DM subjects [4-13]. However, several studies have demonstrated that subcutaneous fat, not visceral fat, is the best predictor of insulin resistance in obese individuals [14-17]. The factors responsible for these inconsistent results have yet to be elucidated. One potential explanation that might account for these discordant reports is the failure to account for differences in gender. Men tend to accumulate adipose tissue in the abdomen, while women tend to accumulate fat in the gluteal-femoral region, in part due to the differences in androgen and/or estrogen action *in vivo *[18,19]. Another potential explanation for the discordant reports might be the failure to account for the differences in the individual body type of the study subject. All previous studies that investigated the association between abdominal fat distribution and insulin resistance evaluated subcutaneous and visceral fat area and/or volume, but these values were not related to the body type of each subject. One would expect that the metabolic impact of visceral and/or subcutaneous fat area *in vivo *would be different between subjects who are 150 cm tall from those who are 180 cm tall, even if both of them have a similar value for visceral and/or subcutaneous fat sectional area. We previously demonstrated that visceral fat area was significantly correlated with peripheral and hepatic insulin resistance, independent of gender. However, in male, but not in female subjects, BMI (body mass index), fat mass, and subcutaneous fat area also were significantly correlated with peripheral and hepatic insulin resistance [20]. Peripheral (muscle) and hepatic insulin sensitivity can be quantitated by measuring the rate of glucose disappearance and appearance, respectively, during the euglycemic hyperinsulinemia clamp and usually expressed per min and per lean body weight. Thus, these parameters of insulin action are to some extent standardized irrespectively of the total body fat mass and body type of each individual subject. Accordingly, some correction of abdominal fat area/volume by the body type of each subject should be instituted when examining the relationship between abdominal fat distribution and insulin sensitivity and circulating metabolic parameters. A number of reports have employed the visceral to subcutaneous fat area ratio (VF/SF ratio) to examine the metabolic impact of visceral fat accumulation. The visceral fat area and/or volume typically correlated more strongly with measured metabolic parameters (insulin action, plasma lipids and cytokine concentrations) than with the VF/SF ratio. However, one could hypothesize that the VF/SF ratio would be a better indicator of visceral fat predominant distribution since it is independent of the individual subject's body type (body weight, body height, BMI, body surface area, total fat mass, total fat free mass).

In the present study, we quantitated the VF/SF ratio using MRI and examined the association between this parameter and peripheral and hepatic insulin sensitivity in male adult type 2 diabetic subjects. We did not include female subjects in order to exclude the effect of age-dependent differences in sex hormone levels, especially pre- and post-menopausal, on the abdominal fat distribution.

## Methods

### Subjects

Thirty-six male patients with type 2 diabetes mellitus were recruited from the outpatient clinic of the Texas Diabetes Institute. Entry criteria included an age = 30–70 years and a fasting plasma glucose concentration (FPG) between 126–260 mg/dl. The patient characteristics of the 36 males are shown in table 1. All patients were in good general health without evidence of cardiac, hepatic, renal or other chronic diseases as determined by medical history, physical examination, and screening blood tests. In all subjects body weight was stable (within ± 2 lbs) for at least 3 months prior to study. Sixteen subjects were taking a stable dose (for at least 6 months) of sulfonylurea drugs and 20 subjects were treated with diet alone. Patients who previously had received insulin, metformin, or a thiazolidinedione were excluded. All subjects gave their written voluntary, informed consent prior to participation. The protocol was approved by the Institutional Review Board of the University of Texas Health Science Center at San Antonio.

**Table 1:** Anthropometric and clinical characteristics

		**(Range)**
Race (MA/C/AA)	22/11/3	
Age (y)	55 ± 2	(32–70)
Duration of Diabetes	6 ± 1	(1–16)
Diet/SU therapy	20/16	
Body Weight (kg)	89 ± 3	(60–119)
Height (cm)	172 ± 1	(156–192)
Body Mass Index (kg/m^2^)	30 ± 1	(22.9–38.9)
Body Surface Area (m^2^)	2.05 ± 0.03	(1.61–2.52)
Fat Mass (kg)	31 ± 2	(13.6–51.5)
Fasting Plasma Glucose (mg/dl)	199 ± 7	(101–265)
Fasting Plasma Insulin (μU/ml)	15 ± 1	(5.5–39.1)
HbA_1c _(%)	9.0 ± 0.2	(6.8–11.9)
Fasting Free Fatty Acid (μEq/L)	589 ± 22	(320–921)
Basal EGP (mg/kg FFM·min)	3.0 ± 0.1	(1.74–4.64)
Clamp EGP (mg/kg FFM·min)	1.2 ± 0.1	(0.2–2.2)
TGD (mg/kg FFM·min)	3.5 ± 0.2	(1.6–7.7)
SSPI	67 ± 2	(44–84)
Clamp EGP × SSPI	76 ± 7	(10–156)
TGD/SSPI	0.05 ± 0.003	(0.02–0.10)
Clamp FFA (μEq/L)	230 ± 13	(118–389)
VF (cm^2^)	159 ± 9	(75–337)
SF (cm^2^)	303 ± 21	(108–651)
VF/SF Ratio	0.59 ± 0.04	(0.17–1.30)
Deep SF (cm^2^)	199 ± 14	(53–418)
Superficial SF (cm^2^)	104 ± 8	(44–232)

MA = Mexican American; C = Caucasian; AA = African American

FFM = fat free mass (kg)

EGP = endogenous glucose production rate

Clamp EGP = endogenous glucose production rate during insulin clamp

SSPI = steady state plasma insulin concentration during insulin clamp

Clamp FFA = plasma free fatty acid concentration during insulin clamp

TGD = total glucose disposal rate during insulin clamp

Clamp EGP × SSPI = product of the Clamp EGP and steady state plasma insulin concentration during insulin clamp (index of hepatic insulin resistance)

TGD/SSPI = TGD divided by SSPI (index of peripheral [muscle] insulin sensitivity)

VF = visceral fat area at L_4–5_

SF = subcutaneous fat area at L_4–5_

### Study Design

Within a 5–7 day interval all subjects (i) measurement of fat free mass and fat mass using an intravenous bolus of ^3^H_2_O; (ii) quantitation of total subcutaneous, superficial subcutaneous, deep subcutaneous, and intraabdominal visceral fat content at L_4–5 _using nuclear magnetic response imaging (MRI); (iii) a euglycemic insulin clamp study in combination with tritiated glucose to examine hepatic and peripheral tissue (muscle) sensitivity to insulin. Fasting plasma concentrations of glucose and free fatty acid and HbA_1c _were measured on the day of the insulin clamp. All studies were done in the postabsorptive state after a 10–12 h overnight fast. Subjects who were taking sulfonylureas stopped their medication 2 days prior to study.

### Fat Free Mass and Fat Mass

At 8 AM (time zero) subjects receive a 100 μCi intravenous bolus of ^3^H_2_O and plasma tritiated water radioactivity was determined at 90, 105, 120 minutes for calculation of fat free mass (FFM) and fat mass (FM) as described previously [8].

### Abdominal Fat Distribution

Intraabdominal visceral and subcutaneous fat depots were measured by MRI, using imaging procedures that have been published previously [21]. Briefly, images were acquired on a 1.9 T Elscint Prestige MRI system, using a T1-weighted spin echo pulse sequence with a TR (repetition time) of 500 msec and a TE (echo time) of < 20 msec. A sagittal localizing image was used to center transverse sections on the line through the space between L_4 _and L_5_. Ten 5.0 mm thick sections were acquired with a gap of 1.0 mm to prevent signal cross-over from adjacent sections. The field of view ranged from 30 to 50 cm, depending on body size. Phase encoding was in the anteroposterior direction to minimize the effects of motion-induced phase artifacts that might otherwise be distributed laterally through a large portion of the abdomen. The field of view was adjusted for body size to insure 2 mm pixel spacing. Signal averaging (four signals averaged) was used to reduce the effect of motion-related artifacts. Additionally, respiratory gating was used to combat motion induced artifacts and to reduce the blurring of fat boundaries in the anterior region of the abdomen. Images were processed using Alice software (Perceptive Systems Inc, Boulder, CO) to determine abdominal subcutaneous and intraabdominal visceral fat areas. The subcutaneous fat area was analyzed by selecting the outer and inner margins of subcutaneous adipose tissue as region of interests (ROIs) from the cross-sectional images and counting the number of pixels between the outer and inner margins of subcutaneous adipose tissue. The abdominal subcutaneous fat area was subdivided into superficial and deep subcutaneous fat areas (SSF and DSF) by identifying the fascial line which demarcates these two fat depots [22]. The visceral (intraabdominal) fat area was determined using histograms specific to the visceral regions. The histograms were summed over the range of pixel values designated as fat by fitting two normal analysis distribution curves to them.

### Euglycemic Hyperinsulinemic Clamp

Insulin sensitivity was assessed with the euglycemic insulin clamp, as previously described [23]. Upon arrival (0800 h.) at the Clinical Research Center, blood for measurement of fasting plasma glucose, HbA_1c _and lipid profile was obtained, and a prime (25 μCi × FPG/100)-continuous (0.25 μCi/min) infusion of ^3^H-3-glucose was started via a catheter placed into an antecubital vein. The tritiated glucose infusion was continued throughout the 7 hour study. A second catheter was placed retrogradely into a vein on the dorsum of the hand, which was then placed in a heated box (60°C). Baseline arterialized venous blood samples for determination of plasma ^3^H-3-glucose radioactivity and plasma glucose, FFA and insulin concentrations were drawn at 150, 160, 170, 175, and 180 minutes after the start of the tritiated glucose infusion. At 180 minutes (1100 h), a prime-continuous infusion of human regular insulin (Novolin, Novo Nordisk Pharmaceuticals, Princeton, NJ) was started at the rate of 40 mU/min·m^2 ^body surface area and continued for 120 min. After initiation of the insulin infusion, the plasma glucose concentration was allowed to drop spontaneously until it reached 90 mg/dl, at which level it was maintained by appropriately adjusting a variable infusion of 20% dextrose. Throughout the insulin clamp, blood samples for determination of plasma glucose concentration were drawn every 5 minutes, and blood samples for determination of plasma insulin and ^3^H-3-glucose radioactivity were collected every 10–15 minutes.

### Assays

Plasma glucose was measured at bedside using the glucose oxidase method (Glucose Analyzer 2, Beckman Instruments Inc., Fullerton, CA). Plasma insulin (Diagnostic Products Corporation, Los Angeles, CA) was measured by radioimmunoassay. HbA_1c _was measured by affinity chromatography (Biochemical Methodology, Drower 4350; Isolab, Akron, OH). Plasma FFA was measured by an enzymatic calorimetric quantitation (Wako Chemicals GmbH, Neuss, Germany). Tritiated glucose specific activity was determined on barium hydroxide/zinc sulfate deproteinized plasma samples.

### Calculations

Under steady state postabsorptive conditions, the rate of endogenous glucose appearance (Ra) was calculated as the ^3^H-3-glucose infusion rate (dpm/min) divided by the steady state plasma ^3^H-3-glucose specific activity (dpm/mg). During the insulin clamp, non-steady conditions prevail and Ra was calculated from Steele's equation [24]. Endogenous glucose production (EGP) was calculated as: Ra minus glucose infusion rate. During the insulin clamp total body glucose disposal (TGD) equals the sum of the residual EGP plus the exogenous GIR. TGD primarily represents insulin sensitivity in skeletal muscle, which is responsible for > 80% of insulin-stimulated glucose disposal during euglycemic hyperinsulinemic clamp studies. We also expressed insulin sensitivity in skeletal muscle as TGD divided by the steady state plasma insulin concentration (SSPI) during the last 30 minutes of insulin clamp (TGD/SSPI), because there are differences in insulin clearance among the individuals. Hepatic insulin resistance was expressed as the residual rate of EGP during the insulin clamp (clamp EGP) and the product of clamp EGP and the steady state plasma insulin concentration (clamp EGP × SSPI). The logic behind this calculation is as follows: (i) under postabsorptive conditions, the majority (~85–90%) of EGP is derived from the liver [25]; (ii) insulin is an inhibitor of hepatic glucose production and increments in the ambient insulin concentration exert a potent inhibitory effect on hepatic glucose output [26], (iii) suppression of hepatic glucose production is impaired under conditions of physiologic hyperinsulinemia (< 100 μU/ml) in obese subjects and type 2 diabetic patients compared with control subjects [26,27], and (iv) there are differences in the steady state plasma insulin concentrations during the insulin clamp because of differences in insulin clearance among study subjects.

Total body water was calculated from the mean plasma ^3^H_2_O radioactivity measured at 90, 105, and 120 min after the intravenous bolus of ^3^H_2_O. Plasma ^3^H_2_O specific activity was calculated assuming that plasma water represents 93% of total plasma volume. Fat free mass (FFM) was calculated by dividing total body water by 0.73 [28].

### Statistical Analysis

Data are given as the mean ± standard error of the mean (SEM). Statistics were performed with StatView for Windows, v 5.0 (SAS Institute Inc., Cary, NC). We used Pearson correlation coefficients to assess the association between fat topography and versus age, BMI, and insulin sensitivity. All results are presented as the mean ± standard error. A p value less than 0.05 was considered to be statistically significant.

## Results

### Correlations between Age, Obesity, and Abdominal Fat Distribution (Table 2)

**Table 2:** Correlation coefficients between age, BMI, and parameters of abdominal fat area.

	**Age**	**BMI**	**BSA**	**FM**	**SF**	**VF**	**VF/SF**	**SSF**	**DSF**
Age									
BMI	-0.03								
BSA	-0.02	0.75 ^a^							
FM	-0.09	0.76 ^a^	0.75 a						
SF	-0.17	0.85 ^a^	0.75 ^a^	0.78 ^a^					
VF	0.35 ^b^	0.55 ^a^	0.46 ^a^	0.46 ^a^	0.28 ^c^				
VF/SF	0.29 ^c^	-0.40 ^b^	-0.40 ^b^	-0.41 ^b^	NA	NA			
SSF	-0.27	0.66 ^a^	0.65 ^a^	0.63 ^a^	0.87 ^a^	0.13	-0.61 a		
DSF	-0.09	0.86 ^a^	0.71 ^a^	0.78 ^a^	0.95 ^a^	0.33 ^b^	-0.62 ^a^	0.69 ^a^	

a: p < 0.01; b: p < 0.05; c: p < 0.1 for regression analysis.

SFF = superficial subcutaneous fat

DSF = deep subcutaneous fat

VF = visceral abdominal fat

SF = subcutaneous abdominal fat

FM = total body fat mass

Age correlated positively with visceral fat area and tended to be positively correlated with V/S ratio. There was no correlation between age and subcutaneous fat area (SF), superficial and deep subcutaneous fat area (SSF & DSF), body mass index (BMI), body surface area (BSA) and fat mass (FM). BMI, BSA and total FM strongly and positively correlated with SF, SSF, and DSF, while BMI, BSA and total FM correlated positively with VF. VF/SF ratio correlated inversely with BMI, BSA and FM, possibly because BMI, BSA and FM correlated more strongly with SF than did VF. VF did not correlate with SF or SSF but correlated significantly with DSF. VF tended to be positively correlated with SF, possibly because VF correlated positively with DSF but not with SSF.

### Relationships between FM, BMI, BSA, VF, SF (SSF, DSF), and VF/SF ratio versus Indices of Peripheral/Hepatic Insulin Resistance, and Free Fatty Acid Concentration (FFA) (Figures 1 &2)

**Figure 1:**
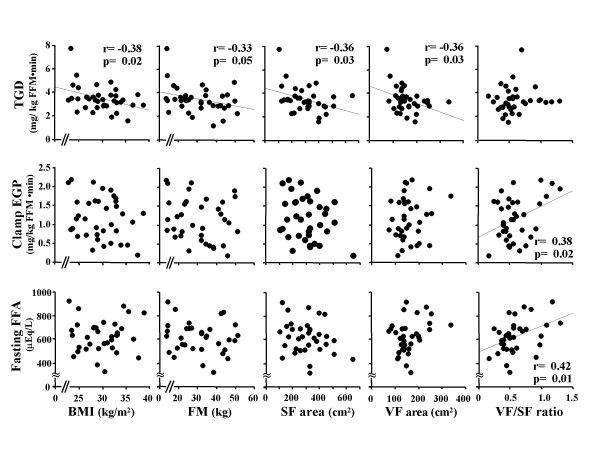
**Relationship between total body glucose disposal (TGD) during the insulin clamp (top), endogenous glucose production during the insulin clamp (middle), and fasting plasma free fatty acid (FFA) concentration (lower) versus body mass index (BMI), total body fat mass (FM), abdominal subcutaneous fat (SF) area and visceral fat (VF) area at the L_4–5 _vertebral level**.

**Figure 2:**
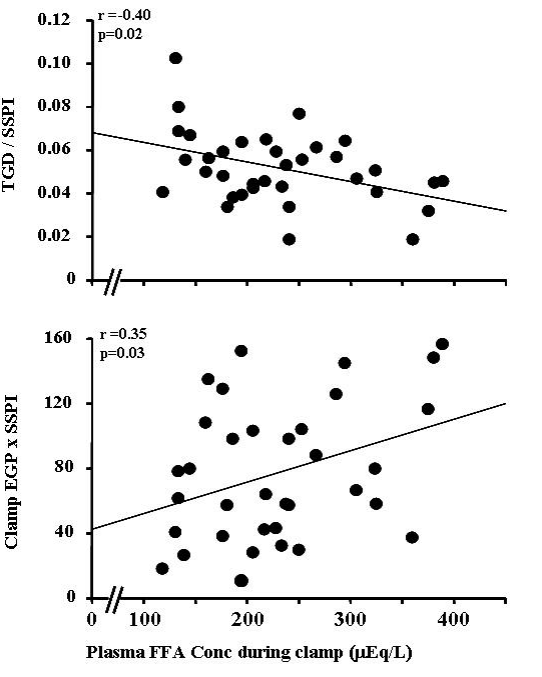
**Relationship between plasma FFA concentration during the insulin clamp versus glucose disposal (TGD) during the insulin clamp divided by the steady state plasma insulin concentration (SSPI) during the insulin clamp (top) and the product of EGP × SSPI during the insulin clamp**.

TGD, an index of peripheral (muscle) insulin sensitivity, correlated inversely with BMI, BSA (r = -0.52, p < 0.01), FM, SF, SSF (r = -0.33, p = 0.05), DSF (r = -0.34, p = 0.04) and VF. TGD/SSPI during the insulin clamp similarly correlated with BMI (r = -0.38, p = 0.02), BSA (r = -0.46, p < 0.01), FM (r = -0.35, p = 0.03), SF (r = -0.37, p = 0.02), DSF (r = -0.40, p = 0.01), and VF (r = -0.34, p = 0.04). VF/SF ratio did correlate with TGD or TGD/SSPI. EGP during the insulin clamp (an index of hepatic insulin resistance) correlated positively with VF/SF ratio but not with BMI, BSA, FM, SF, SSF, DSF, or VF. Similarly, EGP × SSPI during the insulin clamp correlated positively only with the VF/SF ratio (r = 0.39, p = 0.02). Log transformed (TGD/SSPI) correlated inversely with BMI (r = -0.42, p = 0.01), BSA (r = -0.55, p < 0.01), FM (r = -0.41, p = 0.01), SF (r = -0.41, p = 0.01), VF (r = -0.33. p = 0.04), DSF (r = -0.43, p = 0.01), but did not with VF/SF ratio and SSF. Log transformed (Clamp EGP × SSPI) positively correlated with VF/SF ratio (r = 0.36, p = 0.03), but did not with BMI, BSA, FM, SF, VF, SSF, DSF. Fasting plasma FFA correlated positively with VF/SF ratio and weakly with VF. No significant correlations were observed between the plasma FFA concentration and BMI, FM, SF, SSF or DSF. During the insulin clamp, the plasma FFA concentration correlated inversely with TGD/SSPI and positively with clamp EGP × SSPI.

## Discussion

As reviewed in the introduction, controversy remains concerning the contribution of visceral versus subcutaneous fat accumulation to the development of insulin resistance in obesity and type 2 diabetes mellitus (T2DM). A number of previous publications have examined the relationship between peripheral (muscle) and/or hepatic insulin resistance versus the visceral or subcutaneous fat area L_4–5_, which correlates closely with visceral or subcutaneous adipose tissue volumes calculated from multiple scans [29]. These studies have demonstrated the presence of significant associations between insulin resistance and abdominal subcutaneous and visceral fat area/volume, but they did not take into consideration differences in body type of the subjects. Failure to do so could explain some of the discordant reports concerning correlations – or lack thereof – between abdominal fat distribution and insulin resistance. In the present study, we have reexamined whether abdominal visceral fat accumulation correlates with peripheral (muscle) and hepatic insulin resistance in male T2DM subjects, using the visceral to subcutaneous fat area (VF/SF) ratio which provides information about visceral and subcutaneous fat accumulation, independent of the individual's body type.

**Figure 3:**
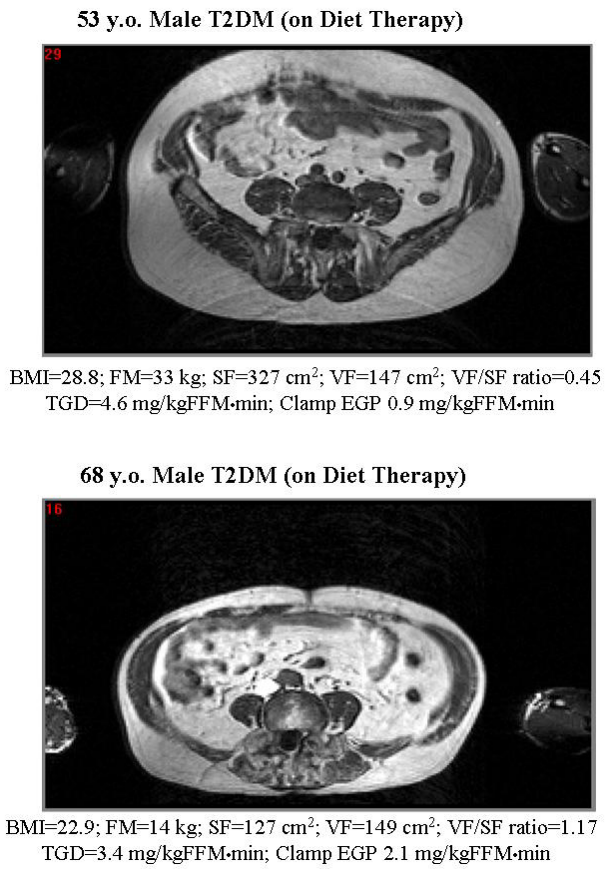
**Transverse cross-sectional magnetic resonance image at the L_4–5 _vertebral level in a study subject whose visceral to abdominal subcutaneous fat (VF/SF) ratio was increased (bottom) or decreased (upper)**.

In this cross-sectional study, we demonstrated that (i) age is positively correlated with visceral fat area (VF), independent of BMI, fat mass (FM), and subcutaneous fat area (SF) (Table 2). This observation is consistent with previous publications [30,31]; (ii) BMI and FM simple indicators of general obesity were more strongly and positively correlated with SF (including superficial and deep SF) than they were with VF, leading to the inverse correlation of VF/SF ratio with BMI, FM and SF (Table 2). These results are consistent with those of Smith et al [32] and the fact that visceral fat (omentum and mesenteric fat) comprises only ~20% of total body fat in man [29]; (iii) BMI, FM, SF, SSF, DSF, and VF all were inversely correlated with peripheral (muscle) insulin sensitivity in male subjects with T2DM, while the VF/SF ratio did not correlate with the peripheral insulin sensitivity. These results are consistent with some previous studies in male subjects [6,14,15], although most prior publications have shown that visceral fat accumulation is correlated with insulin resistance [4-13]. However, one should be cautious in interpreting these previous reports, including our own, because the values of FM, SF (SSF and DSF) and VF were evaluated without consideration of the individual body type of the subject. One would expect that the metabolic impact of visceral and/or subcutaneous fat area *in vivo *would be different between subjects who are 150 cm tall from those who are 180 cm tall, even if both of them have a similar value for visceral and/or subcutaneous fat sectional area. As an index of body type, body weight and/or height, body surface area, and body mass index can easily be measured. These indices, however, never have been used to correct the SF and VF area and/or volume in previously published papers. In our study the VF/SF ratio did not correlate with peripheral insulin resistance. Our results suggest that accumulation of both abdominal subcutaneous and visceral fat, independent of which regional fat depot predominates, is related to peripheral (muscle) insulin resistance in male subjects with T2DM. In contrast, only the VF/SF ratio, and not other parameters of fat mass/distribution, correlated with impaired suppression of EGP during the insulin clamp and with the product of Clamp EGP × SSPI – indices of hepatic insulin resistance. These results indicate that peripheral (muscle) insulin resistance is related to the accumulation of both abdominal subcutaneous and visceral fat (upper-body obesity), while hepatic insulin resistance is more closely correlated with the accumulation of visceral fat, independent of body type, in male T2DM patients (Figure 3).

Adipose tissue is a complex and highly active endocrine organ that secretes multiple adipocytokines including leptin, IL-6, TNFα, resistin, adiponectin, MCP-1 and FFA [33,34]. Adipocyte enlargement, as observed in obesity, is associated with dysfunctional fat cells which over-express and secrete excessive amounts of leptin, IL-6, TNFα, resistin, MCP-1 and FFA while under secreting adiponectin [35,36]. Altered secretions of these adipocytokines have been implicated in the development of insulin resistance in obesity and T2DM [37]. Although there are differences in the expression/production/secretion of the adipokines between subcutaneous and visceral adipose tissue, they are expressed in both fat depots altered production and/or release from either fat depot can lead to altered circulating adipokine levels and impaired insulin action in peripheral tissues (primarily muscle). Our results demonstrate that increased plasma FFA levels during the insulin clamp correlated inversely with peripheral (muscle) insulin sensitivity (TGD/SSPI) and positively with hepatic insulin resistance (Clamp EGP × SSPI). Plasma concentrations of other adipocytokines were not measured in this study. Jensen et al. reported that elevated plasma FFA concentrations in upper-body obesity arose from upper-body subcutaneous fat and increased abdominal SF and VF areas were positively correlated with abdominal subcutaneous adipocyte size [38,39]. However, we failed to find a significant association between the plasma FFA concentration during the insulin clamp and SF (both SSF and DSF), VF or total FM. One could speculate that these discordant results are explained by the failure of Jensen et al [38,39] to take into account differences in the body type of the individual subjects.

With respect to relationship between visceral fat accumulation and hepatic insulin resistance (impaired insulin-induced suppression of glucose production at liver), three scenarios have been proposed [34,40]: (i) altered release of adipocytokines into the portal vein by an expanded mass of visceral adipose tissue; (ii) the relative inability of subcutaneous adipose tissue to act as a protective metabolic sink because of its inability to expand (like lipodystrophy) or because it already has become hypertrophied, dysfunctional and insulin resistant, leading to accumulation of fat at undesired site such as liver, skeletal muscle, pancreas, and heart ("ectopic fat deposition"); (iii) visceral adipocyte resistance to the antilipolytic effect of insulin leads to an increased portal vein delivery of FFA to the liver. Scenarios (i) and (ii) would be expected to be also closely related to peripheral (muscle) insulin resistance, while scenario (iii) more likely would be related to hepatic insulin resistance. In the present study, VF/SF ratio correlated positively with the fasting plasma FFA concentration, although this concentration does not necessarily reflect the portal vein FFA concentration. In a previous publication, we demonstrated that only the VF area correlated positively with accelerated gluconeogenesis, but not with glycogenolysis. This observation is consistent with the concept that increased delivery of FFA from an expanded visceral adipose tissue mass to the liver enhances gluconeogenesis and causes hepatic insulin resistance [41].

In the present study, there were several limitations. First, the number of the study subjects is small and the subjects are not homogenous as far as age and the duration of diabetes. Second, this study does not have healthy control subjects. Therefore, a further investigation using a large population with age- and the duration of diabetes-adjustment to be performed for obtaining a precise evaluation of VF/SF ratio in relation to peripheral and hepatic insulin resistance in T2DM.

## Conclusion

The present results demonstrate that obesity per se (i.e. higher levels of total body fat mass and BMI), independent of subcutaneous or visceral fat accumulation, is related to peripheral (muscle) insulin resistance in male type 2 diabetic patients. In contrast, dominant accumulation of visceral adipose tissue as opposed to increased subcutaneous fat, independent of the individual body type, is related to hepatic insulin resistance in male type 2 diabetic patients. Lastly, our results indicate that it is important to evaluate, not only the abdominal fat area/volume, but also the VF/SF ratio which provides a measure of abdominal fat distribution, i.e., predominancy of SF or VF, independent of the body type of the study subject.

## Abbreviations

VF/SF ratio: intraabdominal visceral to abdominal subcutaneous fat area ratio; TGD: total body glucose disposal; EGP: endogenous glucose production; SF: abdominal subcutaneous fat area; VF: visceral fat area; MRI: magnetic resonance imaging; SSPI: steady state plasma insulin concentration during the insulin clamp; TGD/SSPI: TGD divided by SSPI; BMI: body mass index; FM: total fat mass; T2DM: type 2 diabetes mellitus; FFM: fat free mass; TR: repetition time; TE: echo time; SSF: superficial subcutaneous fat; DSF: deep subcutaneous fat; FFA: free fatty acids; Ra: rate of endogenous glucose appearance; GIR: glucose infusion rate; Clamp EGP: EGP during the insulin clamp.

## Competing interests

The authors declare that they have no competing interests.

## Authors' contributions

YM conceived the study, designed the study experiment, performed the experiments, analyzed data, interpreted results and wrote the manuscript. RAD conceived the study, designed the study experiment, interpreted the results, participated in the writing and critically revised the manuscript. Both authors read and approved the final manuscript.
